# A case-control association study and family-based expression analysis of the bipolar disorder candidate gene *PI4K2B*

**DOI:** 10.1016/j.jpsychires.2009.05.004

**Published:** 2009-12

**Authors:** Lorna M. Houlihan, Andrea Christoforou, Margaret I. Arbuckle, Helen S. Torrance, Susan M. Anderson, Walter J. Muir, David J. Porteous, Douglas H. Blackwood, Kathryn L. Evans

**Affiliations:** a Medical Genetics Section, Molecular Medicine Centre, The University of Edinburgh, Western General Hospital, Crewe Road, Edinburgh EH4 2XU, UK; b Division of Psychiatry, Royal Edinburgh Hospital, The University of Edinburgh, Edinburgh EH10 5HF, UK

**Keywords:** Bipolar disorder, Chromosome 4p15, *PI4K2B*, Phosphatidylinositol pathway, Association, Expression studies

## Abstract

Bipolar disorder, schizophrenia and recurrent major depression are complex psychiatric illnesses with a substantial, yet unknown genetic component. Linkage of bipolar disorder and recurrent major depression with markers on chromosome 4p15–p16 has been identified in a large Scottish family and three smaller families. Analysis of haplotypes in the four chromosome 4p-linked families, identified two regions, each shared by three of the four families, which are also supported by a case-control association study. The candidate gene *phosphatidylinositol 4-kinase type-II beta* (*PI4K2B*) lies within one of these regions. *PI4K2B* is a strong functional candidate as it is a member of the phosphatidylinositol pathway, which is targeted by lithium for therapeutic effect in bipolar disorder. Two approaches were undertaken to test the *PI4K2B* candidate gene as a susceptibility factor for psychiatric illness. First, a case-control association study, using tagging SNPs from the *PI4K2B* genomic region, in bipolar disorder (*n* = 368), schizophrenia (*n* = 386) and controls (*n* = 458) showed association with a two-marker haplotype in schizophrenia but not bipolar disorder (rs10939038 and rs17408391, global *P* = 0.005, permuted global *P* = 0.039). Second, expression studies at the allele-specific mRNA and protein level using lymphoblastoid cell lines from members of the large Scottish family, which showed linkage to 4p15–p16 in bipolar disorder and recurrent major depression, showed no difference in expression differences between affected and non-affected family members. There is no evidence to suggest that *PI4K2B* is contributing to bipolar disorder in this family but a role for this gene in schizophrenia has not been excluded.

## 1 Objectives

Genetic evidence for psychiatric illness, including bipolar disorder, schizophrenia and related phenotypes to chromosome 4p15–p16, has been established in linkage, population and affected individual studies as detailed previously (Le Hellard et al., 2007). Haplotype analysis of chromosome 4p15–p16 using four families with linkage to that region, highlighted two areas in the 20 Mb linked region, where three of the four linkage regions overlap, namely region B and region D (Le Hellard et al., 2007). These regions have been supported by association analysis in the Scottish population (Christoforou et al., 2007). Region D appeared an attractive region for further study because of the strength of the linkage evidence from a Welsh family with schizophrenia and schizoaffective disorder (LOD 2, (Asherson et al., 1998)) and a large family from the United States of Ashkenazi Jewish origin with major mental illness including bipolar disorder and schizophrenia (LOD 3.2, (Detera-Wadleigh et al., 1999)). Of the 13 known genes in region D (Le Hellard et al., 2007), *PI4K2B* has the strongest case as a candidate gene from biological and neuropharmacological evidence. In addition, the aforementioned association study identified SNP rs10939038, which lies in the same linkage disequilibrium (LD) block as *PI4K2B*, as a potentially important variant in schizophrenia cases (allele *P* = 0.006, odds ratio (OR) = 1.314, 95%CI: 1.08–1.59) (Christoforou et al., 2007).

*PI4K2B* is a phosphatidylinositol 4-kinase and a member of the phosphoinositide (PI) signal transduction pathway. Its primary function is the phosphorylation of phosphatidylinositol to generate phosphatidylinositol 4-phosphate (Balla et al., 2002; Wei et al., 2002). The PI signalling pathway is a target for the mood stabilising drugs, lithium and sodium valproate (Berridge et al., 1989; Farmer et al., 2007; Kato, 2007).

This study examined *PI4K2B* as a possible genetic risk factor in bipolar disorder or schizophrenia, by two approaches. First, a case-control association study tested association of additional markers in the *PI4K2B* region in 368 cases with bipolar disorder, 386 cases with schizophrenia and 458 controls from the Scottish population. Second, RNA and protein expression analysis of *PI4K2B* was conducted with lymphoblastoid cell lines from members of the large Scottish family, which showed linkage to the chromosome 4p15–p16 region (Blackwood et al., 1996).

## 2 Materials and methods

### 2.1 Case-control association study

#### 2.1.1 Sample

The cohort consisted of DNA from individuals with bipolar disorder (368; 160 males and 208 females), schizophrenia (386; 276 males and 110 females) and controls (458; 237 males, 218 females and three unknown). Details of this sample have been previously reported (Christoforou et al., 2007). The study was approved by the Multi-Centre Research Ethics Committee for Scotland. The sample comprised individuals contacted through the inpatient and outpatient services of hospitals in South East Scotland. Diagnoses according to DSM-IV criteria were based on information from an interview with the patient using the Schedule for Affective Disorders and Schizophrenia-Life time version (SADS-L) were reached by consensus between two trained psychiatrists. Controls from the same region were recruited through the South of Scotland Blood Transfusion Service and from hospital staff. Control subjects were drawn from the same population in South East and South Central Scotland. The majority (>80%) were recruited through the Scottish National Blood Transfusion service. Although the blood donors were not screened by interview for personal or family history of psychiatric illness, donors are only allowed to donate blood if they are not currently on medication and had no chronic illness. The remaining controls were recruited from the local population and from hospital staff. These controls were briefly screened by interview to exclude anyone currently on medication or with a history of treatment for psychiatric illness.

#### 2.1.2 SNP Selection

SNP genotype data from 30 CEPH trios, 100 kb upstream and downstream of the *PI4K2B* genomic region (24,745,440–24,986,687 bp, NCBI build 35) from HapMap Phase II (January 2006) (http://www.hapmap.org) was uploaded to Haploview version 3.2 (Barrett et al., 2005). Tagging SNPs were selected as previously described (Christoforou et al., 2007), with one exception that haplotypes were tagged down to the 5% level for finer coverage of the region.

#### 2.1.3 Genotyping

Genotyping was performed with pre-designed Taqman assays on demand or assays by design from Applied Biosystems using the ABI PRISM 7900HT sequence detection system. The seven SNPs were successfully genotyped with an average locus success rate of 95% (range: 89–99%) in a total number of 1212 individuals (93% sample success rate). Further descriptive information on the SNPs is available in Supplementary Table 1.

#### 2.1.4 Association analysis

Power calculations showed this study would reach a significance level *α* = 0.01, with >93% power for association of an allele that shows a heterozygote relative risk (multiplicative model) of two, assuming a risk allele frequency of 10% and a lifetime prevalence of 1% in 368 individuals (number of bipolar disorder cases) (Purcell et al., 2003). The power available in our study decreases when more modest relative risks are used (relative risk 1.2–1.5 estimates the available power 18–67% at *α* = 0.01). The standard *χ*^2^ test of independence was used to examine deviations of the 11 SNPs from Hardy–Weinberg equilibrium (HWE) in the control sample (all HWE *P-*values ⩾ 0.001 as Haploview default (Wigginton and Abecasis, 2005); minimum HWE *P* = 0.04). Single-marker analysis was performed using in the *χ*^2^ test of independence (Dudbridge, 2003). Haplotype analysis was performed separately on bipolar disorder and schizophrenia cases with CoCaphase 2.4 (Dudbridge, 2003), using a sliding-windows approach to test all haplotypes of two marker length. Rare haplotypes with a frequency ⩽ 0.05 in both cases and controls were clumped together. Permutation analysis was also performed using CoCaphase.

### 2.2 Expression analysis

#### 2.2.1 DNA samples

Lymphoblastoid cell lines from members of the large Scottish family were grown under standardised conditions, following standard protocols. All cell lines were subjected to the same cell culture and experimental conditions. Genomic DNA (gDNA) and RNA were prepared by the DNeasy Tissue Kit (Qiagen) and RNeasy Mini Kit (Qiagen) respectively. mRNA was reverse transcribed to cDNA with First Strand cDNA Kit (Roche) using random primers. “Quant-iT PicoGreen dsDNA Reagent and Kit” (Molecular Probes) was used to quantify DNA.

#### 2.2.2 Allelic imbalance assay

rs313548 and rs313567 were available as pre-designed Taqman SNP genotyping assays (c_764549, c_764538, respectively) while rs6834255 was a custom designed Taqman SNP genotyping Assay (ABI). A standard curve of dilutions from homozygous lymphoblastoid cell line samples was prepared to evaluate the contribution of each allele in the heterozygote samples; genomic DNA prepared from two homozygote samples (5 ng/μl), as previously published (Pastinen et al., 2004). Ten microgram of mixed gDNA was assessed in a total volume of 5 μl, 2 μl gDNA, 0.25 μl Taqman SNP Genotyping Assay, 2.5 μl Taqman^®^ Universal PCR Master Mix, 0.25 μl dH_2_O. Each homozygote mix was assayed in quadruplicate. A standard curve (linear regression line) was established for the LOG of the fluorescent intensity ratio (FAM^™^/VIC^®^) versus the log of the allele ratio, i.e. the LOG_2_ ratio of the dilution of each homozygote (90:10 = LOG_2_ Ratio is 3.147), as previously published (Lo et al., 2003). For the heterozygote DNA samples, 4 ng cDNA or 10 ng gDNA was amplified; 2 μl cDNA (2 ng/μl) or 2 μl gDNA (5 ng/μl), 0.25 μl Taqman SNP Genotyping Assay, 2.5 μl Taqman^®^ Universal PCR Master Mix, 0.25 μl dH_2_O. Each sample was prepared in quadruplicate and each assay was performed in triplicate from the same RNA preparation. The standard assay was performed in the ABI PRISM 7900HT sequence detection system (Applied Biosystems). From the endpoint read, the ratio of the fluorescence reading from the FAM^™^ reporter dye and VIC^®^ reporter dye was calculated. cDNA values were calculated by the cDNA/gDNA ratio from the mean value of DNA from the same individual.

#### 2.2.3 Quantitative immunoblotting

The Western blotting procedure was performed on 5 μg protein lysates using NuPAGE^®^ Electrophoresis System, as per manufacturer’s instructions with 17-well 4-12% Bis–Tris gels. Densitometry analysis was performed using ImageJ v 1.37 (Abramoff et al., 2004) to quantify the immunoreactive protein in each lane.

## 3 Results

### 3.1 Case-control association study

**Fig. 1:**
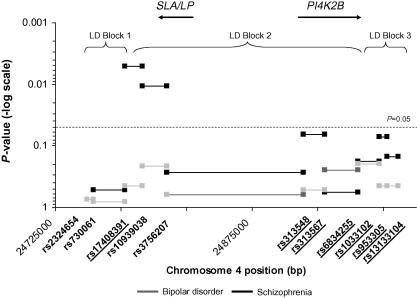
Distribution of global haplotype *P*-values from two-marker sliding window haplotype analysis in *PI4K2B* region. The *x*-axis is the chromosome 4 physical position in base pairs (bp) according to the human genome map of UCSC May 2004, NCBI build 35. The *y*-axis is the *P*-values on a – log scale. The results are shown for bipolar disorder in grey and schizophrenia in black. The nominal significance threshold is indicated with a dashed horizontal line at *P* = 0.05. The LD blocks are indicated by a bracket. The LD map of the region was defined by solid spine of LD (D’ > 0.8) using HapMap CEU trios downloaded on 7th February 2006, phase II b125. The position of genes in this region *PI4K2B* and *SLA/LP* are denoted with black arrows. The markers underlined are the follow-up markers specific to this present study (*n* = 7) and those not underlined were genotyped previously (*n* = 4) (Christoforou et al., 2007). The *P*-value for the first two markers was the same for both bipolar disorder and schizophrenia and overlap so cannot be seen.

Fig. 1 shows sliding-window analysis of two-SNP haplotypes where two haplotypes emerged as significant at the global level in the schizophrenia sample: rs17408391–rs10939038 (global *P* = 0.005, permuted global *P* = 0.039, SE:0.006) and rs10939038–rs3756207 (global *P* = 0.01, permuted global *P* > 0.05). Supplementary Table 2 shows the significant individual haplotype structures and corresponding *P*-values for these global haplotypes. Haplotype rs17408391–rs10939038 spans from LD block 1 to LD block 2, while haplotype rs10939038–rs3756207 falls within LD block 2 as illustrated in Fig. 1. Analysis at the single-marker level showed the only significant single-marker association less than the nominal significant level (*P* = 0.05) was rs10939038 in the schizophrenia cases (*P* = 0.006), which was previously reported (Christoforou et al., 2007) (data not shown). This association withstood the gene-wide permutation testing (1000 simulations) performed for this study (permuted *P* = 0.015, standard error (SE) = 0.0038). No association was detected in either bipolar disorder or schizophrenia at the single-marker level (all single marker *P*-values > 0.05) for the seven additional SNPs, which were selected to increase coverage of the *PI4K2B* region.

### 3.2 Expression studies

**Fig. 2:**
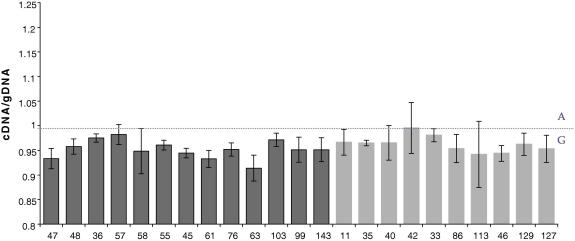
PI4K2B allelic expression at rs313548 between linked haplotype carriers and controls. The heterozygote lymphoblastoid cell line samples are shown on the *x*-axis. The cDNA/gDNA ratio for each sample is shown on the *y*-axis. This ratio is the mean of three assays, each with four replicates, calculated as intensity FAM/intensity VIC. The error bars show 95% Confidence Intervals. The dark grey bars represent samples with the *PI4K2B* linked haplotype that are heterozygote at this SNP. The light grey bars represent samples without the linked haplotype. A dotted line is drawn at one, which represents equal expression from both alleles.

**Fig. 3:**
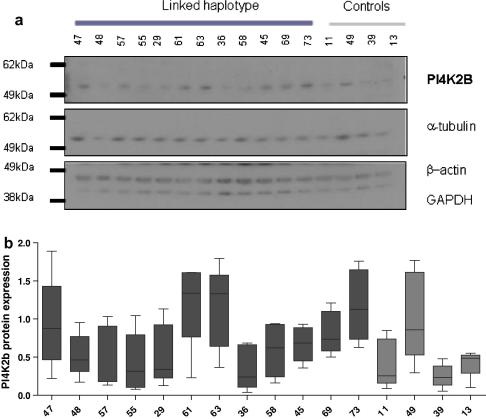
No evidence for a PI4K2B protein expression difference between linked haplotype carriers and controls. PI4K2B protein expression (a) was detected at 55 kDa in 5μg protein preparations from lymphoblastoid cell lines (Minogue et al., 2001). This is an example Western blot of five Western blots performed. Detection of α-tubulin, β-actin and GAPDH at 50, 42 and 40 kDa, respectively were used as a loading control. Protein expression levels were measured by densitometry (Abramoff et al., 2004). PI4K2B expression level was normalised against the mean expression level from the protein loading controls. The box and whiskers plot (b) shows the five-number summary of five replicate gels; lower quartile, median, upper quartile, and largest observation. The dark grey bars represent individuals with a linked haplotype and light grey bars represent married-in control individuals.

*PI4K2B* expression was measured at the RNA and protein level in lymphoblastoid cell lines from members of a large Scottish family. Certain members of the family have a defined haplotype on chromosome 4p15–p16, referred to as “the linked haplotype” that segregates with the majority of cases of bipolar disorder and recurrent major depression, as described previously (Le Hellard et al., 2007). RNA expression of *PI4K2B* was assessed in lymphoblastoid cell lines by allele-specific quantitative RT-PCR using three SNPs by Taqman technology comparing relatives with and without the “linked haplotype”. Estimation of allelic imbalance from standard curves was performed as previously described (Pastinen et al., 2004). Based on the standard curves, we found that deviation from equal expression could reliably be detected from both alleles (50:50) at a 80:20 allele ratio at rs313567, a 90:10 allele ratio at rs313548 and a 70:30 allele ratio at rs6834255, respectively. The allelic imbalances observed for the heterozygote cDNA samples did not reach the detection threshold (data not shown). To detect a more sensitive difference, gDNA measurements from heterozygotes were incorporated, which represented equal expression from each allele, as a control, as previously performed (Bakker et al., 2007). Fig. 2 shows the allelic expression at rs313548 between linked haplotype carriers (*n* = 13) and controls (*n* = 10). There was no difference in allele expression of rs313548 between individuals with the *PI4K2B* linked haplotype and those who do not have the haplotype (*t*-test *P* = 0.28). Furthermore, there was no difference in allele expression between individuals with the *PI4K2B* linked haplotype and those who do not have the haplotype for rs6834255 (*t*-test *P* = 0.98) and rs313567 (*t*-test *P* = 0.36) (data not shown). PI4K2B protein expression was detected in lymphoblastoid cell lines derived from the large Scottish family: Twelve samples with a “linked haplotype” and a psychiatric diagnosis [bipolar disorder (*n* = 4), recurrent major depression (*n* = 4), no symptoms (*n* = 4)] and founder individuals as controls (*n* = 4). Fig. 3 shows no evidence for a *PI4K2B* protein expression difference between linked haplotype carriers and controls in five replicate western blot experiments (*t*-test *P* > 0.1).

## 4 Discussion

*PI4K2B* was a worthy candidate gene for bipolar disorder in many respects. Positional evidence from linkage and association analyses have implicated this genomic region on chromosome 4p15–p16. Genetic evidence has also suggested other members of the phosphoinositide signalling pathway as susceptibility factors for bipolar disorder, schizophrenia and recurrent major depression (Kato, 2007) and components of the phosphoinositide pathway are inhibited by lithium, a therapeutic agent in bipolar disorder (Berridge et al., 1989). Indeed a recent consortium has been initiated to investigate all genes involved in the lithium pathway as candidate genes for psychiatric illness (Schulze et al., in press) (www.ConLiGen.org). Our study contributes to the genetic basis of psychiatric illness research by adopting a candidate gene study with two approaches; (i) an association study in 368 bipolar disorder, 386 schizophrenia and 458 Scottish participants and (ii) an expression study in lymphoblastoid cell lines from bipolar disorder and recurrent major depression members of a large Scottish family.

This study identified association between a haplotype in *PI4K2B*, which withstood gene-wide permutation testing, and schizophrenia, but not bipolar disorder. This improved haplotype association, relative to the original single-marker finding, may better tag the actual susceptibility variant and thus narrow the target region. If replicated, the association results may reflect association with any feature within the large >240 kb genomic region investigated, in particular *PI4K2B* or a neighbouring gene *SLA/LP*. Previous evaluation of population structure showed no evidence of population stratification in the control group, suggesting that this is unlikely to be the cause of any spurious association due to population stratification (Christoforou et al., 2007).

This study of *PI4K2B* expression levels has shown no difference in lymphoblastoid cell line samples from bipolar disorder family members with the “linked haplotype” and those without. This was demonstrated at the allele-specific level by Taqman assays and at the protein level by quantitative immunoblotting techniques. The advantage of the Taqman method was the sensitivity obtained by comparing the linked haplotype to a series of different control alleles within the one family, using a well-validated method (Bakker et al., 2007; Pastinen et al., 2004). This method could reveal the presence of cis-acting regulatory variants, which may contribute to susceptibility for bipolar disorder. *Cis*-acting variation has been reported to account for 25–35% of inter-individual differences in gene expression throughout the human genome (Pastinen and Hudson, 2004). Three *PI4K2B* SNPs were used to measure gene expression levels, one of which was an intronic SNP to measure heteronuclear RNA as previously described (Pastinen et al., 2004). Initially, standard curves of homozygote gDNA dilutions were created, to look for a deviation from the expected 1:1 allelic ratio in cDNA. Despite optimisation attempts, the standard curves did not achieve the required sensitivity to detect small differences. However, the standard curves were useful to show that a gross deviation in allele-specific expression was not present. A second method, which compared expression in cDNA to that seen in gDNA from heterozygous samples, proved more sensitive. Indeed, in the future it may be useful to investigate lymphoblastoid cell lines from schizophrenia patients for *PI4K2B* expression differences due to the above association study evidence.

The limitations of this expression study are the sensitivity of the technical procedures, the reduced power from the limited number of heterozygote family members with viable lymphoblastoid cell lines available and whether lymphoblastoid cell lines effectively model pathogenic processes in the brain (Gladkevich et al., 2004; Tsuang et al., 2005). Studies have shown that whole blood does share significant gene expression similarities with multiple central nervous system tissues (Sullivan et al., 2006). There is also evidence to suggest that gene expression profiles and functional effects in blood lymphocytes do correlate with psychiatric illness (Gladkevich et al., 2004; Iwamoto et al., 2004; Marazziti et al., 2006; Tsuang et al., 2005; Vawter et al., 2004).

This study showed that there was no direct evidence for a role of *PI4K2B* in susceptibility to bipolar disorder from expression studies in lymphoblastoid cell lines. Nevertheless, this candidate gene still merits further investigation to assess its potential role in psychiatric illness, because of the significant case-control association study result that withstood permutation testing.

## Conflict of interest

All authors declare that they have no conflicts of interest.

## Contributors

Lorna M. Houlihan, Kathryn L. Evans and David J. Porteous designed the study and wrote the protocol. Lorna M. Houlihan managed the literature searches, undertook the statistical analysis and performed the expression experiments. Andrea Christoforou helped in design and interpretation of the association study. Margaret I. Arbuckle, Helen S. Torrance and Susan M. Anderson helped with sample preparation and molecular biology techniques. Douglas H. Blackwood and Walter J. Muir ascertained the samples. Lorna M. Houlihan wrote the first draft of the manuscript. All authors contributed to and have approved the final manuscript.

## Role of funding source

Funding for this study was provided by the Wellcome Trust Grant 072874; the Wellcome Trust had no further role in study design; in the collection, analysis and interpretation of data; in the writing of the report; and in the decision to submit the paper for publication.
