# Supplementary material for: A case-control association study and family-based expression analysis of the bipolar disorder candidate gene PI4K2B
Source: J Psychiatr Res. 2009 Dec;43(16-3):1272–7. doi: 10.1016/j.jpsychires.2009.05.004 (PMC2789249; doi:10.1016/j.jpsychires.2009.05.004)
Supplement: Supplementary data [file mmc1.doc]

| **Number** | **Name** | **Physical Position (bp)** | **LD Block** | **Major Allele** | **Minor Allele** | **MAF** | **genotypes Cases** | **Genotypes Controls** |
| --- | --- | --- | --- | --- | --- | --- | --- | --- |
| **1** | **rs2324654** | **24,748,920** | **1** | **C** | **T** | **0.29** | **71/322/347** | **36/195/227** |
| **2** | **rs730061** | **24,754,154** | **1** | **C** | **G** | **0.26** | **45/280/421** | **35/166/252** |
| **3** | **rs17408391** | **24,777,853** | **1** | **G** | **C** | **0.21** | **35/229/481** | **19/122/238** |
| **4** | **rs10939038** | **24,790,933** | **2** | **C** | **T** | **0.47** | **139/366/240** | **98/217/129** |
| **5** | **rs3756207** | **24,809,633** | **2** | **T** | **C** | **0.19** | **24/214/508** | **20/132/303** |
| **6** | **rs313548** | **24,913,660** | **2** | **A** | **G** | **0.24** | **47/256/437** | **23/132/222** |
| **7** | **rs313567** | **24,930,264** | **2** | **T** | **C** | **0.17** | **15/193/537** | **14/97/266** |
| **8** | **rs6834255** | **24,955,589** | **2** | **G** | **A** | **0.17** | **15/196/534** | **14/95/263** |
| **9** | **rs1033102** | **24,971,539** | **3** | **G** | **C** | **0.3** | **72/306/359** | **31/165/183** |
| **10** | **rs953305** | **24,977,611** | **3** | **T** | **C** | **0.42** | **143/376/226** | **73/170/133** |
| **11** | **rs13133104** | **24,986,084** | **3** | **C** | **T** | **0.1** | **8/139/571** | **8/59/294** |

**Supplementary Table 1** **Descriptive data on *PI4K2B* association study SNPs.** This is a list of the eleven SNPs used in the case-control association study. Their physical position is according to NCBI build 35 of the May 2004 UCSC genome browser. Their position within the LD blocks around PI4K2B is noted. The markers tag all haplotypes >5% frequency in HapMap population. The minor allele frequency (MAF) was measured from the Scottish control population. The genotypes for all cases (bipolar disorder and schizophrenia) and controls are reported.

| rs17408391 | rs10939038 | rs3756207 | Freq Cases | Freq Controls | Individual *P*-value (χ^2^) | Global *P*-value (LRT) |
| --- | --- | --- | --- | --- | --- | --- |
| G | C | - | 0.42 | 0.33 | 0.0007 | 0.005 |
| G | T | - | 0.38 | 0.46 | 0.006 | 0.005 |
| - | C | T | 0.41 | 0.34 | 0.003 | 0.01 |
| - | T | T | 0.40 | 0.46 | 0.007 | 0.01 |

**Supplementary Table 2** **Individual haplotypes significantly associated with schizophrenia**. Freq is the frequency and LRT is the log ratio test.
